# Herpes Simplex Virus 1 Deregulation of Host MicroRNAs

**DOI:** 10.3390/ncrna4040036

**Published:** 2018-11-23

**Authors:** Maja Cokarić Brdovčak, Andreja Zubković, Igor Jurak

**Affiliations:** Laboratory for Molecular Virology, Department of Biotechnology, University of Rijeka, R. Matejčić 2, HR-51000 Rijeka, Croatia; maja.cokaric@biotech.uniri.hr (M.C.B.); azubkovic@biotech.uniri.hr (A.Z.)

**Keywords:** miRNAs, host–pathogen interaction, HSV-1, latency, productive replication, antiviral innate immune response, apoptosis

## Abstract

Viruses utilize microRNAs (miRNAs) in a vast variety of possible interactions and mechanisms, apparently far beyond the classical understanding of gene repression in humans. Likewise, herpes simplex virus 1 (HSV-1) expresses numerous miRNAs and deregulates the expression of host miRNAs. Several HSV-1 miRNAs are abundantly expressed in latency, some of which are encoded antisense to transcripts of important productive infection genes, indicating their roles in repressing the productive cycle and/or in maintenance/reactivation from latency. In addition, HSV-1 also exploits host miRNAs to advance its replication or repress its genes to facilitate latency. Here, we discuss what is known about the functional interplay between HSV-1 and the host miRNA machinery, potential targets, and the molecular mechanisms leading to an efficient virus replication and spread.

## 1. Introduction

Throughout millions of years of co-evolution with their hosts, viruses have been successfully avoiding defense mechanisms and, at the same time, hijacking and harvesting the products of the host’s metabolism to thrive. Interestingly, ancient cells have invented an ingenious mechanism based on small RNAs to defeat viruses, which has later evolved into an elaborate microRNA (miRNA) system (reviewed in [1,2]), also successfully embraced by viruses. In brief, miRNAs are a large class of small non-coding RNAs that govern post-transcriptional repression of mRNAs in animals, plants, and viruses (reviewed in [3]). miRNAs derive from precursor transcripts (pri-miRNAs) that, at least in one region, form a typical hairpin structure, which is recognized by a heterotrimeric complex called Microprocessor containing RNase III enzyme Drosha and its processivity factor DGCR8 [4]. Microprocessor releases the hairpin (≈60 nucleotides) called pre-miRNA, which is transferred to the cytoplasm by Exportin 5/RAN-GTP [5] for further processing by another RNase III enzyme Dicer. Dicer in association with TRBP cuts the hairpin to generate a miRNA duplex with 2–3 nt 3′ overhangs. The duplex containing the miRNA paired to its star strand (miRNA*) is loaded into an Argonaute (Ago) protein, and while miRNA* will be released and degraded, the other strand (miRNA) will form a mature silencing complex [6]. The miRNA in the silencing complex will pair with target transcripts, leading to their degradation and/or translational repression. The target recognition depends on the miRNA seed sequence (nucleotides 2–8) and the complementary pairing with target mRNA, usually within the three prime untranslated region (3′UTR) of messenger RNAs (mRNAs). In animals, miRNAs have important roles in all biological processes, from embryogenesis to development and homeostasis, and have been found to be deregulated in many diseases [7]. Each miRNA can regulate many targets, and it has been estimated that more than 60% of human protein-coding genes are under selective pressure to maintain pairing with miRNAs [8]. A remarkable functional flexibility, non-immunogenicity, small size, and fast evolution (one nucleotide change dramatically shifts the target repertoire) give miRNAs an advantage over proteins in the viral gene regulation [9,10]. Thus, it is not surprising that many viruses have been found to encode miRNAs and/or exploit host miRNAs to their advantage. Currently, there are more than 40 viruses encoding miRNAs (vmiRNAs) listed in miRBase (the main miRNAs database; release 22: March 2018), and this number represents only a fraction of the reported studies and confirmed viral miRNAs (vmiRNAs). Among these, large DNA viruses of the *Herpesvirales* order dominate the list, which can be explained by their biological properties and the accessibility to the miRNA-biogenesis machinery. Herpesviruses establish a lifelong dormant infection (latency) within their hosts, which requires a precise balancing between the maintenance of latency and the responsiveness to reactivation stimuli. miRNAs are potent regulators of gene expression, which might enable a tight regulation of immunosurveillance and/or factors that stimulate reactivation (i.e., miRNAs confer the robustness of the on/off switch).

Clustering of vmiRNAs within the region of a virus genome associated with the active gene expression during latent infection and an abundant expression of vmiRNAs specifically during the latent program strongly support this concept (reviewed in [11,12,13]). Although the negative regulation of virus gene products is counterintuitive from the perspective of virus replication, there are many studies showing that the main role of latent vmiRNAs is to limit the expression of gene products that are important for the productive infection (e.g., virus-encoded trans-activators of gene expression) and preventing host defense mechanisms [9,10] to facilitate the establishment of latency and further spread. For example, Kaposi’s sarcoma-associated herpesvirus (KSHV) miR-K12-9, expressed in latency, regulates the major lytic switch the replication and transcription activator (RTA) protein, which controls the virus reactivation from latency, and inactivation of miR-K12-9 leads to an increased reactivation [14]. Similarly, human cytomegalovirus (HCMV) miR-UL112-1 targets the crucial viral trans-activating gene *IE72* [15], suggesting its possible roles in latency. Yet, not all herpesviruses express miRNAs. Varicella zoster virus (VZV) and Simian varicella virus (SVV), its non-human primate counterpart, have not been found to encode miRNAs [16,17,18], suggesting that other viral and/or host gene products could compensate for their roles. Indeed, a study by Pan et al. (described in detail below) has shown that the host miRNA miR-138, which is expressed in herpes simplex virus 1 (HSV-1) latently infected cells, regulates the expression of ICP0, a viral protein that enhances the reactivation [19], and promotes the establishment of latency.

In contrast to the abovementioned herpesviruses, which use miRNAs to limit their replication, many viruses use host miRNAs to facilitate their productive infection by direct association with miRNAs or by deregulating host miRNAs to generate, for the replication, favorable conditions (reviewed in [20]). The first characterized direct miRNA–virus genome interaction was between hepatitis C virus (HCV) and the liver-specific miR-122. Binding of miR-122 to the 5′UTR of the genome upstream of the internal ribosomal entry site (IRES) enhances protein translation and virus accumulation [21,22] and it is essential for an efficient virus replication. Interestingly, the recruitment of Ago proteins to the 5′UTR stabilizes the RNA genome and prevents its decay [23,24]. In contrast to such positive regulation, a negative regulation by host miRNAs would be under a strong selection pressure and, thus, unexpected in RNA viruses. Indeed, Heiss et al. have introduced target sites for brain-enriched miRNA in the 3′ non-coding region of the flavivirus genome, which strongly reduced virus neurovirulence in the mature mouse central nervous system (CNS). However, in highly permissive suckling mice, virus rapidly reverted to a neurovirulent phenotype accumulating mutations in miRNA binding sites [25], indicating that such target sites might be unsustainable. However, Trobaugh et al. have shown that the hematopoietic cell-specific miRNA miR-142-3p potently restricts the replication of the mosquito-borne North American eastern equine encephalitis virus (EEEV) in myeloid-lineage cells by directly targeting virus genome. This restriction led to a limited induction of immunity, which in turn facilitated replication of the virus and neurological disease manifestations. Moreover, binding of miR-142-3p was essential for the efficient virus infection of mosquito vectors [26]. Similarly, murine cytomegalovirus (MCMV) uses a highly abundant transcript *m169* to decoy two cellular miRNAs, miR-27a and miR-27b, which are subsequently rapidly degraded. A mutant virus no longer able to target miR-27a/b shows a significant attenuation in multiple organs [27,28], indicating an antiviral function of miR-27. The depletion of miR-27 was also observed in herpesvirus-saimiri-infected cells by the highly abundant non-coding RNA HSUR [29]. Thus, clearly, the repressive host miRNA target sites can be evolutionarily conserved within the virus genomes and transcripts, but only if this repression confers some advantage to the virus life cycle.

In contrast to direct pairing of host miRNAs with viral genomes or transcripts, there are numerous studies showing that viruses can dramatically alter the miRNAome of the infected cells, consequently deregulating cellular factors involved in host defense, control of cell death, and virulence (reviewed in [20]), some of which are described below. Based on the described examples, it is important to note that viruses are the most diverse biological entity, and, therefore, it is not surprising that the interplay between viruses and miRNAs is also by far more diverse than a canonical understanding of the miRNA functions. Taken together, viruses additionally prove their resourcefulness and fascinating ability to adapt.

In this review, we briefly summarize the biology of Herpes simplex virus 1 (HSV-1), a prototype of α-herpesviruses, and the current understanding of HSV-1-encoded miRNAs and their functions. In the second part, we focus on the exploitation of host miRNAs by HSV-1 to facilitate latency; suggested functions of the host miRNAs deregulated during the virus infection; and the molecular mechanisms by which the virus triggers expression of the specific host miRNAs. Finally, we discuss experimental challenges in addressing the functions of miRNAs in HSV-1 infection and perspectives for further research in the field.

## 2. Herpes Simplex Virus 1 and miRNAs

HSV-1 is a widely disseminated and an important human pathogen, and one of most intensively studied viruses. HSV-1, similar to all herpesviruses, prospers by having its life cycle in two very distinct phases: productive and latent. The virus initially infects epithelial cells, usually in the oro-nasal region and less commonly of the genital mucosa, which initiates its productive infection. In this phase, the virus abundantly expresses its genes in a coordinated cascade of gene expression (first immediate early (IE), followed by early (E) and late (L) genes) and replicates its DNA, resulting in new virus progeny and spread. Newly replicated viruses can gain access to the nearby innervating neurons and move by retrograde axonal transport to the nucleus of neurons resident in peripheral ganglia. The virus delivers its DNA, which is rapidly chromatinized, and the activity of genes across the entire genome is largely suppressed [30]. In latently infected neurons, the only abundantly expressed transcripts are long non-coding RNAs (lncRNAs) and miRNAs arising from the latency-associated transcripts region (LAT). The major lncRNA is a very stable intron (1.5/2 kb) processed from a long transcript spanning the entire LAT region [31]. The molecular mechanisms that control establishment, maintenance, and reactivation from latency are still poorly characterized, and the exact function of LAT has not been determined. However, the functions in repressing the productive gene expression and enhancing reactivation, inhibition of cell death and host defense, and heterochromatinization of virus genome have been assigned to it [32,33,34,35,36,37,38,39,40,41]. The more recent discovery of HSV-1 miRNAs led to a paradigm shift in understanding the molecular mechanism governing latency and provoked a great interest in researching the roles of HSV-1 miRNAs [42,43,44]. It is important to note that HSV-1 shares many biological properties with HSV-2, a closely related virus that is widely known as the causative agent of genital herpes, including a set of the functional miRNA homologs [43]. Although we will focus on the miRNA interaction of HSV-1 and its host in this review, many of the biological phenomena can be attributed to HSV-2 as well.

### 2.1. HSV-1 miRNAs

HSV-1-encoded miRNAs have been predicted, together with other herpesviruses, by Pfeffer et al. [45]; however, the laboratories of Donald M. Coen and Bryan Cullen have provided the first experimental evidence of an HSV-1-encoded miRNA, miR-H1, which is abundantly expressed in productive infection [42] and miR-H2-H6 in latency [44]. Since then, several groups have contributed to the discovery of HSV-1- and HSV-2-encoded miRNAs, and, currently, evidence has been provided for at least 20 miRNA encoding loci named miR-H1–H8, H11–H18, and H26–H29 (the gaps indicate HSV-2 miRNAs with no identified homologs in HSV-1) [18,43,44,46,47,48,49,50]. Most of the abundantly expressed HSV-1 miRNAs are located within the LAT region and strongly depend on the activity of the LAT promoter in a mouse model [51]. The exact roles of most of the HSV-1-encoded miRNAs are unknown, and have been investigated in only a few instances. For some miRNAs, the genomic locus is very indicative of their function, i.e., some are encoded antisense to important viral genes and, thus, completely complementary to their transcripts. For example, miR-H2, H7, and H8 are antisense to the ICP0 transcript, an important IE transcriptional activator. Similarly, miR-H3 and H4 are antisense to ICP34.5, the main neurovirulence factor. However, although the ICP0 transcript is an obvious target of miR-H2, and although several lines of evidence indicate their interplay (e.g., confirmed specific targeting [44] and interaction identified by ribonucleoside-enhanced crosslinking and immunoprecipitation (PAR-CLIP) [52]), virus mutants of the HSV-1 strain KOS lacking the miR-H2 expression did not show a significant difference in the expression of ICP0 or replication, compared to wild-type (wt) in cultured cells [52,53] or in a mouse model [53]. Then again, a mutant virus with disrupted expression of miR-H2 in the virulent wt strain (McKrae) showed an increased expression of ICP0 during productive infection and increased virulence and rate of reactivation [54,55]. These results are puzzling and, on the one hand, might be explained by the differences between virus strains and the experimental conditions. On the other hand, host and viral miRNAs might have redundant functions, and thus multiple-miRNA mutants might be required to observe more exaggerated phenotypes. Also, the function of miRNAs, i.e., fine tuning of gene expression, might be difficult to test using standard latency and reactivation assays with a relatively low power to capture subtle differences.

Similarly to the miR-H2–ICP0 relation, mutations in miR-H3 or -H4 showed a modest but significant effect on the replication of the virus in a cell-specific manner [52].

It is of note that mutations in the miR-H2 or miR-H4 counterparts in HSV-2 did not identify roles of these miRNAs for the HSV-2 infection either [56]. miR-H7 and H8 lie antisense to the first intron in *ICP0* and are, thus, unlikely to regulate its expression; however, their interplay has not been investigated. ICP4, another important HSV-1 IE transcriptional activator, has been indicated to be a target for regulation by miR-H6, yet the biological relevance of this regulation is still to be investigated [44,57]. In contrast to miR-H2, H3, or H4, most of the HSV-1 miRNAs do not have an obvious target, and, thus, it is conceivable that this target might be host transcripts.

miR-H1 is a unique HSV-1 miRNA expressed abundantly in productive infection and not present in latency and, importantly, encoded at the same locus as miR-H6. This suggests its function in productive infection/reactivation, potential targeting of host transcripts, and even the possibility of a functional interplay between miR-H1 and H6 [58]. Indeed, miR-H1 has been shown to target alpha-thalassemia/mental retardation syndrome X-linked (ATRX), an effector of intrinsic immunity and ND10 component, which contributes to other HSV-1 mechanisms in depletion of this protein [59]. The relevance of ATRX targeting for the HSV-1 infection is still unknown. More recently, Zheng et al. have proposed that ubiquitin protein ligase E3 component n-recognin 1 (Ubr1) is a target of miR-H1, which might contribute to neurodegeneration by interfering with the ubiquitin-proteasome degradation pathway [60]. The molecular mechanism of this regulation is rather puzzling, since miR-H1 is not expressed in latency; however, it might be induced during reactivation. In addition, miR-H1 has been predicted to target genes involved in endocytic and intercellular trafficking pathways affecting immune defense [61]. Additionally, Naqvi et al. have shown an impaired phagocytosis and aberrant cytokine secretion in primary human macrophages (Mφ) transfected with miR-H1, probably by targeting *SORT1* transcript. Moreover, they have observed that miR-H1 deregulates a number of host miRNAs in Mφ and primary oral keratinocytes, which also have predicted roles in endocytosis [61]. Taken together, these studies indicate a complex network of possible multiple interactions, but the exact roles of miR-H1 in infection are yet to be investigated.

Interestingly, in a targeted screen, Enk et al. have investigated whether HSV-1 miRNAs downmodulate NK receptor ligands, and shown that miR-H8 reduced the expression of two NKG2D ligands ULBP2 and ULBP3, the expression of the 2B4 ligand CD48, and viral restriction factor tetherin by targeting phosphatidylinositol glycan anchor biosynthesis class T (PIGT), a member of the protein complex involved in the glycosylphosphatidylinositol (GPI) anchoring pathway essential for the presentation of proteins on the cell surface [62]. Consequently, this downmodulation led to reduced NK-dependent killing [62]. Unfortunately, miR-H8 targets exclusively human PIGT, and, thus, this regulation cannot be tested in a mouse model. Taken together, it is not yet possible to explain the biological relevance of all the abovementioned interactions; however, the pieces of the puzzle are coming together increasingly fast, and, evidently, miRNAs are important for HSV-1 infection.

### 2.2. HSV-1 Deregulation and Interaction with Host miRNAs

To enable efficient virus replication, HSV-1 dramatically alters host cell metabolism, including massive changes in transcriptome and proteome. Modulation of host miRNAs (miRNAome) by HSV-1 and the roles of host miRNAs in HSV-1 infection have been studied extensively throughout the last decade, and a large body of evidence has accumulated. Some host miRNAs have been found to directly regulate virus gene products important for productive infection and, thus, regulate virus entry into latency, whereas others were found deregulated to promote an efficient productive replication by targeting different factors involved in host defense and survival. Below, we will describe some recent evidence of the complex interaction between HSV-1 and host miRNAs.

#### 2.2.1. Direct Targeting of Host miRNAs to HSV-1 Transcripts

**Figure 1:**
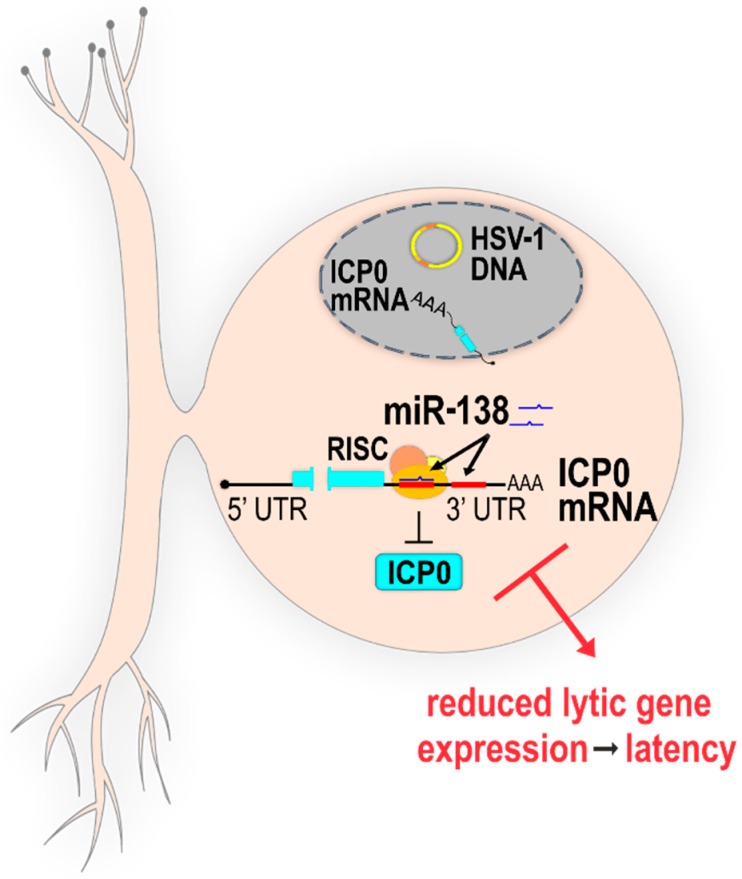
Host miR-138 targets herpes simplex virus 1 (HSV-1) *ICP0* messenger RNA (mRNA) and promotes latency. A schematic representation of host-neuron-specific miR-138 regulating an important productive replication viral protein ICP0. miR-138 in association with RNA-induced silencing complex (RISC) binds to two microRNA (miRNA) target sites within the three prime untranslated region (3′UTR) of the HSV-1 *ICP0* transcript, leading to decreased protein levels of ICP0, and reduced overall lytic gene expression to facilitate latency. Figure adapted from Pan et al. [19].

As mentioned above, the negative regulation of virus replication by direct targeting of host miRNAs to virus transcript is rather unexpected due to a strong selection for loss of such target sites. However, herpesviruses are opportunistic pathogens that infect their hosts in a rather limited fashion, establish “undetectable latency”, and occasionally reactivate and spread. Thus, utilizing every mechanism that would restrict potentially aggressive productive infection should be expected, and there is an increasing body of evidence showing that a tightly controlled virus infection represents an advantage, and miRNAs might have a crucial role in these processes. Recently, Pan et al. have identified two miR-138 target sites within the 3′UTR of HSV-1 *ICP0* mRNA, and one such site within the HSV-2 *ICP0* mRNA, which indicated an evolutionarily conserved functional role. miR-138 is a highly conserved miRNA abundantly expressed in neuronal cells and important for neuronal stem cell proliferation and differentiation, and frequently found downregulated in various cancers (reviewed in [63]). This led the authors to a hypothesis that miR-138 might limit the expression of ICP0 in neurons, thus repressing the productive infection to facilitate latency [19]. Indeed, in addition to a standard transfection assay in which miR-138 can reduce the expression of its target, Pan et al. have shown evidence of RNA-induced silencing complex (RISC) binding to the *ICP0* transcript in association with miR-138 by PAR-CLIP in cells infected with HSV-1. To explore the biological relevance of miR-138–ICP0-mediated regulation, they have generated viruses with mutated miR-138 binding sites. Interestingly, although endogenous miR-138 can reduce the expression of ICP0 in productively infected cells in culture, the mutant virus replicated with similar kinetics to wt in all cells tested. However, the experiments in a mouse model gave a dramatically different phenotype. During the acute phase of infection, mutant and wt virus replicated comparably and waned at the same pace. However, later in the infection (day 7 post infection (p.i.); i.e., towards the establishment of latency) the authors have observed increased levels of *ICP0* and other productive replication gene transcripts, including elevated levels of viral DNA, compared to wt, indicating that the mutant virus is debilitated for the establishment of latency. Moreover, the mutation in the *ICP0* miR-138 binding site resulted in significantly increased morbidity and mortality in mice. Curiously, after the establishment of latency (32 days p.i.), the expression levels of *ICP0*, *LAT*, and other markers of virus infection were comparable between mutant virus and wt, and both viruses reactivated equally efficiently from latency. These results can be explained by challenging experimental approaches and the robustness of assays with the sensitivity below the detection of meaningful differences. The authors speculated that HSV-1 has evolved to utilize the host miRNA to gain the selective advantage of increased latency, which in turn increases the ability of the virus to spread throughout the population [19] (Figure 1).

It is important to note, as described above, that the *ICP0* transcript also contains two binding sites for the virus-encoded miR-H2, and that mutations of miR-H2 also showed an increased neurovirulence in strain McKrae [54,55], but the phenotype was not statistically significant in strain KOS [53]. It would be interesting to learn if mutations in both miR-H2 and binding sites for miR-138 would additionally accentuate the observed phenotype. Nonetheless, although the mysterious molecular mechanisms controlling the HSV-1 latency are becoming even more complicated, it is now clear that miRNAs have an important role in these processes.

#### 2.2.2. Indirect Effects of Host miRNAs on HSV-1 Infection

**Figure 2:**
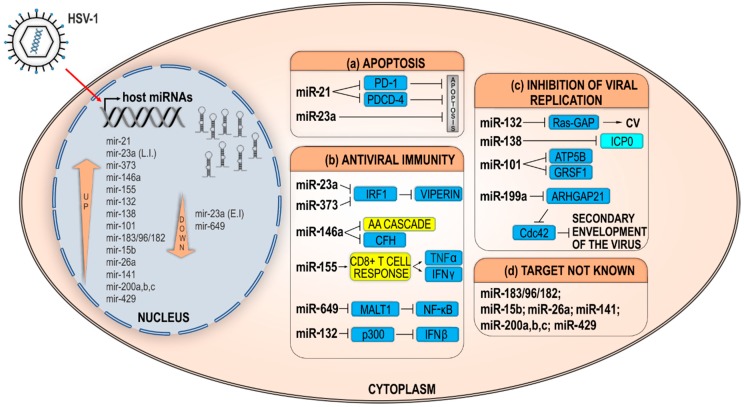
HSV-1 deregulates host miRNAs. A schematic representation of reported deregulated host miRNAs in HSV-1 infection and miRNAs with pro/antiviral functions. HSV-1 infects cells and triggers massive changes in host cell miRNAome. Many miRNAs have been found to be upregulated (arrow up within the nucleus) or downregulated (arrow down in the nucleus). These miRNAs regulate different host (blue boxes) or viral transcripts (light blue box) with functions in (**a**) regulation of apoptosis, (**b**) antiviral immunity, (**c**) inhibition of viral replication, and (**d**) miRNAs with targets not known depicted in separate boxes. During early infection (EI), miR-23a is downregulated, while later in the infection (LI) miR-23a is upregulated. The exact targets of miR-155 and miR-146a have not been identified; however, they regulate host immune response (yellow boxes) by regulating T-cell differentiation and the arachidonic acid cascade (AA cascade) pathway, respectively. Upregulated miR-132 activates Ras by removing Ras-GAP, leading to corneal neovascularization (CV). Neuron-specific miR-138 regulates virus protein ICP0. Arrows indicate positive regulation. PD-1: Programmed cell death protein 1; PDCD-4: Programmed cell death protein 4; IRF1: Interferon regulatory factor 1; CFH: Complement factor H; TNFα: Tumor necrosis factor alpha; IFNβ/γ: Interferon beta/gamma; MALT1: Mucosa-associated lymphoid tissue lymphoma translocation gene 1; NF-κB: Nuclear factor kappa-light-chain-enhancer of activated B cells; Ras-GAP: Ras–glyceraldehyde-3-phosphate; ICP0: Infected cell polypeptide 0; ATP5B: ATP synthase subunit beta; GRSF1: G-rich sequence factor 1; ARHGAP21: Rho GTPase-Activating Protein 21; Cdc42: Cell division control protein 42 homolog.

HSV-1 has been found to modulate more than a dozen host miRNAs to target host virulence factors enabling an efficient virus replication, and a vast number of such interactions is yet to be identified. The deregulated host miRNAs can indirectly impact the HSV-1 replication through various mechanisms, which, although intersected, can roughly be grouped into three categories: (a) miRNAs that modulate factors important for the replication, (b) miRNAs that prolong cell survival, and (c) miRNAs targeting factors of immunity. Below, we will summarize the work of many groups that have contributed to this aspect of the complex HSV-1 biology (Figure 2 and Table 1).

##### Host miRNAs Prolonging Cell Survival and/or Weakening Host Defense Mechanisms

Virus infection is recognized by elaborate host defense mechanisms, many of which overlap and lead to a less-permissive state for virus replication or programmed cell death. Apoptosis is form of programed cell death that is vital for normal cell turnover and the removal of old, damaged, or infected cells. Expectedly, miRNAs were also found to indirectly control apoptosis by targeting different genes involved in cell survival, and many such miRNAs have been found to be deregulated in cancer [64]. The importance of these mechanisms for limiting virus infection can be appreciated from the fact that all viruses encode inhibitors of these pathways. For example, HSV-1 encodes at least seven gene products that have been shown to inhibit apoptosis at some phase of infection (reviewed in [65]).

Recently, Ru et al. have found that HSV-1 infection strongly induces miR-23a in productively infected HeLa cells [66], which coincides with the depletion of interferon regulatory transcription factor 1 (IRF1). miR-23a is well-known oncomiR (a miRNA with a transformation potential) with roles in cell proliferation, development, immunity, and, in particular, apoptosis by modulating many different targets, including IRF1 [67]. In addition, Ru et al. showed that miR-23a-mediated depletion of IRF1 can result in lower levels of radical S-adenosyl methionine domain-containing 2 (RSAD2)/viperin, suggesting its role in limiting virus infection [68]. Interestingly, the gammaherpesvirus KSHV encodes a number of miRNAs, including miR-K9, which targets growth arrest DNA damage-inducible gene 45 beta (*GADD45B*) and prevents cell cycle arrest and apoptosis [69], and miR-K3, a miRNA that shares targets with miR-23a (i.e., a functional homolog), including genes involved in the regulation of apoptosis and caspase-3 and -7 [70,71]. In addition, overexpression of miR-23a has been shown to inhibit Porcine reproductive and respiratory syndrome virus (PRRSV) by directly targeting virus RNA and inducing an IFN response [72]. miR-21, another miRNA that has been well-characterized as a suppressor of apoptosis, has been found to be strongly upregulated in various cells in a mouse model of Behcet’s disease after infection with HSV-1; however, the relevance for the HSV-1 infection has not been revealed [73]. On the other hand, Xie et al. showed further evidence for the importance of the HSV-1–miRNA–IRF1 feedback regulation in infection [74]. Using miRNA arrays, they have shown that miR-373, which is involved in cell growth, apoptosis, and immune response, was one of the most upregulated miRNAs in HeLa cells after infection with HSV-1. It is of note that they have not observed a significant change in the expression levels of miR-23a. Also, they have found an increased expression of miR-373 in serum from patients with herpetic gingivostomatitis compared to healthy control. In an effort to investigate the biological relevance of this induction, they show that transient overexpression of miR-373 slightly increased the virus yield, whereas the inhibition of miR-373 decreased the yield by more than 10×, indicating a proviral role for miR-373. Moreover, they have shown that miR-373 targets *IRF1* mRNA directly and, thus, limits the expression of many proteins involved in interferon response, including the expression of IFN-α, IFN-β, protein kinase R (PKR), and 2′,5′-oligoadenylate synthetase (OAS) [74], and, thus, facilitates the replication of HSV-1. Similar to HSV-1, miR-373 has been found to facilitate the replication of PRRSV [75], hepatitis B virus (HBV) [76], and HCV [77], suggesting a broad role of this miRNA in promoting virus replication through negative regulation of the IFN signaling pathway.

miR-146a is one of the miRNAs most frequently found to be deregulated in virus infections, and was the first host miRNA shown to be upregulated in HSV-1 infection [83]. The expression of miR-146a is responsive to stimulation by pathogens and immunomodulatory cytokines, such as TNFα and IL-1β, and it regulates the innate, immune, and inflammatory response and other antiviral pathways by targeting many genes, including TNF receptor-associated factor 6 (*TRAF6*) and interleukin-1 receptor-associated kinase 1 and 2 (*IRAK1* and *IRAK2*) (reviewed in [87,88]). Hill et al. have observed the upregulation of miR-146a, but not the closely related brain-enriched miR-132 (mentioned below), in primary human neuronal-glial (HNG) cells infected with HSV-1 [83]. They linked the elevated expression of the miR-146a with the concurrent downregulation of complement factor H (CFH), a known target of this miRNA and repressor of the complement signaling cascade, and suggested that this regulation loop might also contribute to the first-line antiviral host defense mechanism [83]. This observation was later confirmed by Majer et al. when analyzing global changes in miRNA expression during HSV-1 encephalitis (HSVE) in mice. The authors have observed that a number of immuno-modulatory miRNAs were upregulated, including miR-146a, the miR-183/96/182 cluster, miR-155, and the miR-200 family [85]. It is rather puzzling to note that some of these miRNAs are proinflammatory (e.g., miR-155), whereas others suppress inflammation (e.g., miR-146a), which can be explained by a different response of various cells to the infection, which might have contributed differently in a global analysis. Indeed, Majer et al. have shown evidence that the proinflammatory miR-155 was largely overexpressed in cells with a microglia phenotype. It has been previously shown that miR-155 knockout (KO) mice are more susceptible to HSV-1 ocular infection and dissemination in the nervous system due to a diminished function of CD8 T cells [89], strongly suggesting its protective role. However, in wild-type mice, ocular infection with HSV-1 leads to inflammation and overexpression of miR-155, mainly in macrophages and CD4(+) T cells [84], which strongly contributes to stromal keratitis (SK) and corneal vascularization (CV). Thus, miR-155 KO mice or mice treated in situ with miR-155 inhibitors were more resistant to herpes SK [84], which identifies miR-155 as a potential target for inhibition to control not only herpetic keratitis, but other immune-related adverse diseases, such as autoimmunity. There is a vast interest in researching miR-155 in virus replication, yet, from the virus replication perspective, the best evidence of its importance comes from KSHV and Marek’s disease virus (MDV), which encode orthologs of miR-155 to influence maturation and expansion of B-cells [10,90,91,92].

Similar to miR-146a and miR-155, miR-132 has been found to be implicated in the replication of many viruses, including HSV-1. miR-132 has roles in neuronal function and development, angiogenesis, and innate immune response, and has been associated with Alzheimer’s disease [93]. Initially, Lagos et al. have shown that KSHV infection of lymphatic endothelial cells (LECs), as well as HSV-1 or HCMV infection of monocytes, induces the expression of miR-132 early in the infection, and in the absence of virus gene expression [82]. The induction of miR-132 is required for efficient KSHV replication and depends on the phosphorylation of the cAMP response element-binding protein (CREB) by mitogen/stress-activated protein kinases. Transcriptional coactivator EP300 (p300), a protein that associates with CREB and is essential for the initiation of antiviral immunity [94], was the revealed target of miR-132; i.e., in conclusion, the virus induces a negative feedback loop to facilitate its replication and impairs host defense. In the context of HSV-1 infection and similar to miR-155, miR-132 has been found to be strongly upregulated in corneas of mice after HSV-1 infection [81], depending on the VEGF or IL-17 signaling. This signaling leads to neovascularization and SK lesions, which can be prevented by miR-132 antagomirs [81].

In contrast to all of the abovementioned miRNAs, which were found to be upregulated in HSV-1 infection, recently, Zhang et al. have reported that miR-649 was downregulated in productively infected HeLa cells in an multiplicity of infection (MOI)-dependent manner [78]. There is relatively limited knowledge about the function or targets of miR-649; however, roles in carcinogenesis have been attributed to it [95,96,97]. Zhang et al. have shown that miR-649 facilitates HSV-1 replication through direct targeting of 3′UTR of mucosa-associated lymphoid tissue lymphoma translocation gene 1 (*MALT1*) in both HeLa and Hep-2 cells. The paracaspase MALT1 is an essential component of nuclear factor κB (NF-κB) signaling that plays a key role in innate and adaptive immunity. Downregulation of MALT1 results in evasion of both innate and adaptive immune responses through inhibition of the NF-κB pathway [78]. Interestingly, miR-649 levels were downregulated after HSV-1 infection in HeLa cells, and its downregulation may play a role in limiting HSV-1 replication through a negative feedback loop. Although investigators have proposed a molecular mechanism by which miR-649 directly targets *MALT1* and in that way facilitates replication of HSV-1, further studies are necessary to confirm the significance of this mechanism in virus–host interaction.

##### Host miRNAs Modulate Host Factors Required for Efficient HSV-1 Replication

In an effort to identify virulence factors important for the virus infection, Zhang et al. have observed that overexpression of miR-101 reduced the replication of HSV-1 in HeLa cells, whereas the presence of a miR-101 inhibitor slightly increased the replication of the virus compared to a control inhibitor. In addition, they have observed that miR-101 is upregulated in productively infected HeLa cells [79]. This observation led them to hypothesize that miR-101 might be a part of an inducible defense mechanism targeting host factors important for virus replication. Indeed, they have identified ATP synthase subunit beta (ATP5B), a subunit of the mitochondrial F1 ATP synthase complex crucial for maintaining the energy homeostasis in cells, as a target for the miR-101 regulation. Surprisingly, the overexpression of miR-101, although reducing a vital component of the cells, did not alter the cells viability; nonetheless, these cells were less permissive to HSV-1 infection [79]. More recently, the same group has shown that ICP4, the major transcriptional regulatory protein of HSV-1, binds directly to the miR-101 promoter and induces its expression [80]. In addition, they have validated another target of miR-101, RNA–binding protein G-rich sequence factor 1 (GRSF1), a member of a large family of RNA-binding proteins termed the heterogeneous nuclear ribonucleoprotein F/H protein (hnRNP F/H) family. GRSF1 has been identified as a host virulence factor for many viruses, and it has a role in facilitating protein synthesis by direct binding to viral RNAs [98,99,100]. Similarly, Wang et al. have shown that, in transfection assays, GRSF1 directly binds HSV-1 *p40* (*UL26*) mRNA and enhances its expression. Taken together, Wang et al. have concluded that miR-101 might have a role in repressing GRSF1 to limit virus replication, and to ensure the survival of host cells permitting a persistent HSV-1 infection, similar to what has been described for HSV-1-encoded miRNAs and host miR-138. Notably, in contrast to the upregulation of miR-101 during HSV-1 infection, miR-101 has been found to be among the most downregulated miRNAs in HCMV-infected cells, and linked to regulation of mammalian target of rapamycin (mTOR) signaling components leading to reduced mTOR protein levels, and, thus, affecting mRNA translation and cell growth and survival [101]. HSV-1 and HCMV, although both herpesviruses that share many biological properties, are quite different viruses and, thus, it is not surprising that they might differ in some aspects of their replication.

It is important to mention that miRNAs were successfully used to discover factors and networks important for viral infection, which can be further explored as potential drug targets. In one such screen, Santhakumar et al. have identified miR-199a as a broadly active antiviral miRNA that inhibits the infection of several viruses, including HSV-1. miR-199 regulates multiple pathways essential for the efficient replication of herpesviruses, including PI3K/Akt and ERK/MAPK signaling [102], and targets Rho GTPase-Activating Protein 21 (ARHGAP21), the Cdc42-specific GTPase-activating protein required for normal Golgi function and for the HSV-1 secondary envelopment [103].

#### 2.2.3. The Molecular Mechanism of HSV-1 Induced Upregulation of the miR-183/96/182 Cluster

**Figure 3:**
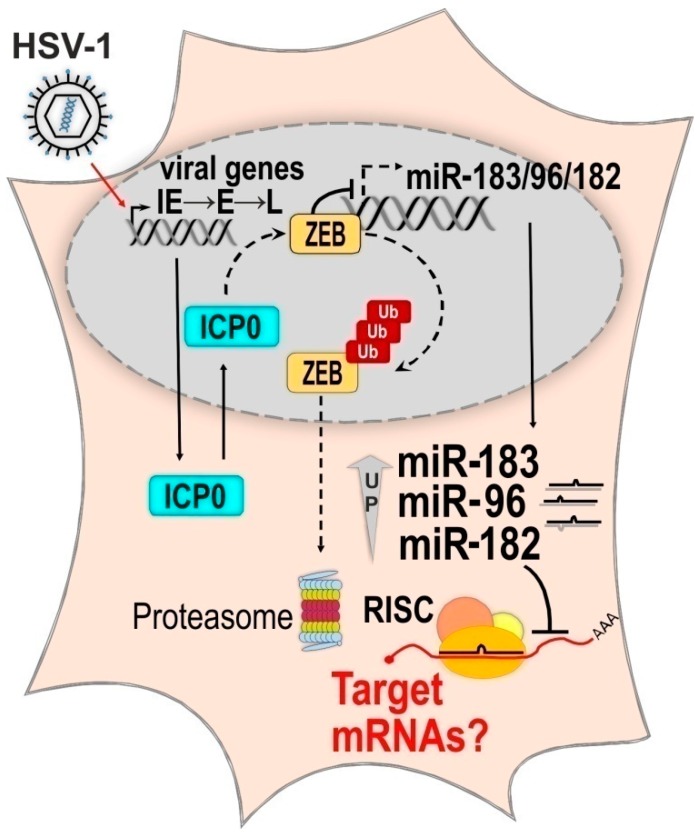
The molecular mechanism of the miR-183/96/182 cluster upregulation in HSV-1 infection. HSV-1 expresses its genes in a controlled cascade of gene expression, first immediate early (IE), early (E), and then late (L) genes. IE protein ICP0 directs host protein Zinc Finger E-Box Binding Homeobox (ZEB) for ubiquitin-dependent proteasomal degradation and de-represses the miR-183/96/182 cluster [86]. The targets of the co-expressed miR-183, miR-96, and miR-182 are unknown. Adapted from Lutz et al. [86].

A vast body of evidence suggests that any virus infection will trigger deregulation of host miRNAs to a different extent [29,104,105,106,107]; however, in only a few instances has the exact molecular mechanism of the observed deregulations been revealed. As described above, many studies have shown that HSV-1 triggers massive changes in host miRNAs, but most of these studies were limited to only one cell type or specific experimental conditions. Recently, Lutz et al., while analyzing the miRNA expression pattern in two different HSV-1 in vitro latency models, have observed that the relative abundance of overall miRNAs does not dramatically change, suggesting that HSV-1 does not cause global changes in host miRNA abundance [86,108]. In addition, they have observed that miR-183, miR-96, and miR-182, which are miRNAs expressed from a single cluster, were significantly upregulated during early times of latency establishment in vitro and during the productive infection in primary cells (human foreskin fibroblasts or rat neurons) but not in transformed cell lines (HeLa, U2OS) [86]. More recently, Majer et al. have found that this cluster is upregulated in brain tissue of mice with acute HSVE, indicating roles for these miRNAs in host defense mechanisms [85]. miRNAs of the miR-183/96/182 cluster are co-expressed and have important roles in stemness, embryogenesis, and development [109,110,111], and are frequently found to be deregulated in cancer and other diseases (reviewed in [112]). Using a battery of virus mutants deficient for the expression of different IE proteins, Lutz et al. have demonstrated that the expression of ICP0 and ICP4 is required for the maximal induction of the miR-183/96/182 cluster during HSV-1 infection [86]. Furthermore, they have shown that the expression of ICP0 in the absence of any other HSV-1 gene product is sufficient to induce the cluster and that is mediated by the E3 ligase function of ICP0. This led the authors to speculate that ICP0 might target a repressor of the miR-183/96/182 transcription for degradation. Indeed, they have identified the consensus binding sequences for the ZEB (Zinc Finger E-Box Binding Homeobox) family of transcription factors in the upstream region of the potential transcriptional start site of the primary transcript of the cluster. Moreover, two members of the family and well-known repressors of the miR-183/96/182 cluster, ZEB1 and ZEB2 [113], have been previously identified as SUMOylated proteins destabilized by ICP0 [114]. Taken together, Lutz et al. provided additional evidence that ICP0 indeed is responsible for the depletion of ZEB1, which in turn coincides with the de-repression of the miR-183/96/182 cluster and the elevated expression of each of the miRNAs (Figure 3). Importantly, HSV-1 is not the only herpesvirus that alters the expression of the miR-183/96/182 cluster or interacts with the ZEB proteins, but rather this interplay represents a broadly conserved mechanism. Similar to HSV-1, HCMV alters the abundance of several host miRNAs during its productive infection cycle, including a strong upregulation of the miR-183/96/182 cluster [115,116]. HCMV does not encode an ICP0 homolog; however, it is well-established that immediate early protein IE1 and tegument protein pp71 are, to an extent, functional counterparts [117] of ICP0; however, it is not known if these proteins modulate the expression of the cluster. Interestingly, high levels of miRNAs of the miR-183/96/182 cluster have also been found to be associated with the Epstein–Barr virus (EBV) type I latency, as compared to barely detectable levels of these miRNAs in the type II or III latency [118,119], in which other sets of miRNAs are expressed at high levels (e.g., miR-146a or miR-155). Oussaief et al. have shown that a single virus protein, latent membrane protein 1 (LMP-1), through phosphatidylinositol 3-kinase (PI3K)/Akt signaling selectively downregulates the expression of the miR-183/96/182 cluster and in turn upregulates the targets of these miRNAs [118]. The roles of ZEB1 and ZEB2 in regulating the EBV latent-lytic switch have been investigated in detail, and it has been shown that these proteins repress the expression of the EBV BLZF1 gene by binding to its promoter. However, only downregulation of ZEB2, but not ZEB1, leads EBV towards reactivation [120,121,122]. It would be interesting to learn if upregulation of miR-183/96/182 would lead to reactivation in the absence of other viral or cellular factors.

## 3. Discussion

The roles of miRNAs in HSV-1 infection are largely unexplored, yet a significant amount of literature has accumulated. We focused on the HSV-1/host miRNAs interactions to illustrate the complexity and the challenging aspects in the field. Based on the presented studies, which are mostly limited to one cell line or specific experimental conditions, it is somewhat difficult to find a consensus on which sets of host miRNAs are reproducibly deregulated in HSV-1 infection and how these interactions relate to infection in vivo.

Nonetheless, there is strong evidence that the manipulation of host miRNAs can affect the outcome of HSV-1 infection [80,82,102]. Thus, it is important to address which of these miRNAs represent a part of a host-triggered defense, which miRNAs are actively deregulated by HSV-1 to facilitate replication, and which of these are deregulated as a consequence of the infection and not biologically meaningful. To answer these questions, more integrative research with different HSV-1 strains and different cell types is needed.

In addition, there are several conceptual problems with addressing the roles of host miRNAs in HSV-1 infection, which are a subject of debate in the field. First, regarding the productive infection, HSV-1 has a relatively short replication time, which inevitably leads to the destruction of the infected cell, and, thus, one can argue that miRNAs might not have a chance to significantly contribute to the regulation of productive infection. Secondly, there are numerous more robust viral functions that intercept and block defense mechanisms of the host (e.g., RNase activity of virion host shutoff (Vhs) protein, ICP0-mediated protein degradation, and encoded inhibitors of apoptosis); therefore, the contribution of miRNAs to these processes during the productive infection could be modest at best [59]. Moreover, the observed changes in host miRNA expression levels are relatively small (2–10×), and only nascent proteins or proteins with a relatively short lifetime would be possibly affected by viral or deregulated host miRNAs. On the other hand, one can argue that the current knowledge is largely based on experiments in cultured cells, and might not reflect the relevance in vivo. It is rather frequent that the phenotype of a mutant virus is detectable only in animal models. A good example is the loss of the pathogenicity of mutant MCMV unable to deplete miR-27 [27]. Furthermore, it is interesting to note that, in contrast to all RNA viruses tested (HIV-1, West Nile virus, yellow fever virus, influenza A virus etc.) that were refractory to depletion of endogenous miRNAs, HSV-1 replication was modestly but significantly decreased in Dicer KO cells [123], indicating that viral or host miRNAs might have a role during the productive infection. It has recently been shown that the function of miRNAs might be limited to the regulation of cytokine response after virus infection [124], and, indeed, most of the host miRNAs were reproducibly found to be upregulated in HSV-1 productive infection and target some aspect of innate immunity [66,74,82,83]. However, it is very challenging to experimentally verify the importance of this regulation, particularly in vivo. Notably, the selected proviral miRNAs might represent a target for novel treatment of HSV-1 infection [81,84,102]. It is important to note that herpesvirus infection is a journey through many stages, from a forceful productive infection through establishment and maintenance of latency to reactivation, and different levels of regulation are needed, and some miRNAs might even have a different role at a different stage of the infection. In other words, when addressing the function of miRNAs, one has to take into account that antiviral activity might represent, at the same time, an antiviral defense that limits productive infection and a selective advantage for the virus to establish latency, similar to what was documented for miR-138 [19]. This additionally hampers the already demanding experimental approaches required to reveal the function of miRNAs. For example, if it occurs, it would be extremely difficult to measure the extent and the outcome of host miRNA deregulation in neurons after virus entry.

In contrast to productive infection, the roles of host and viral miRNAs are easier to perceive in the latent phase of HSV-1 infection, and there is much evidence to support this hypothesis. However, experimental challenges remain, because the latency is largely limited to experiments in animal models. However, the recent advent of various in vitro HSV-1 latency models [108,115,125], which are particularly suitable for high-throughput miRNA analyses, will certainly facilitate this research regardless of the limitation regarding the immunity.

Briefly, to conclude, the miRNA field is still relatively young and the exact molecular mechanisms of how miRNAs contribute to HSV-1 infection have not yet been revealed. However, the field is rapidly developing and one can expect a major contribution in understanding of the complex biology of HSV-1.

## Figures and Tables

**Table 1:** Host microRNA (miRNA) deregulated in herpes simplex virus 1 (HSV-1) infection and their targets.

miRNA	Up-/Downregulated	Target	Possible Roles	Model	References	Cellular Process	
**miR-23a**	Down- then Upregulated	IRF1	Inhibition of innate immune response and cell survival	HeLa	[66]		APOPTOSIS
**miR-649**	Downregulated	MALT1	Inhibition of innate and adaptive immune response	HeLa	[78]		
**miR-101**	Upregulated	ATP5B	Blocking DNA packaging and capsid maturation	HeLa	[79]	INHIBITION OF VIRAL REPLICATION	
	Upregulated	GRSF1	Inhibition of viral protein synthesis	HeLa	[80]		
**miR-132**	Upregulated	Ras-GAP	Immuno-inflammatory response leading to neovascularization and stromal keratitis lesions	Murine corneas	[81]	ANTIVIRAL IMMUNITY	
	Upregulated	p300	Inhibition of innate immune response	Monocytes (THP-1 cell line)	[82]		
**miR-146a**	Upregulated	Complement factor H	Evasion of HSV-1 from the innate immune response	Human neuronal-glial cells	[83]		
**miR-373**	Upregulated	IRF1	Inhibition of innate immune response	HeLa and patients with herpetic gingivostomatitis	[74]		
**miR-155**	Deficiency	SOCS1	Regulation of T cell differentiation	In vivo mouse model of herpes simplex encephalitis	[84]		
	Upregulated	Unknown	-	In vivo acute viral encephalitis model—mouse brain	[85]	TARGET NOT KNOWN	
**miR-183/96/182**	Upregulated	Unknown	-	Primary fibroblasts and neurons	[86]		
**miR-15b, miR-26a, miR-141, miR-183/96/182, miR-200a, b, c, miR-429 ^1^**	Upregulated	Unknown	-	In vivo acute viral encephalitis model—mouse brain	[85]		

^1^ All of these miRNAs are shown to be upregulated during HSV-1 infection, and syndecan-2 (Sdc2) has been shown to be a possible cellular target regulated by miR-96, miR-141, miR-183, and miR-200c.
